# Performance of home-based self-tonometry (iCare HOME (TA022)) for measuring intraocular pressure among healthy and glaucoma patients

**DOI:** 10.12688/f1000research.123104.1

**Published:** 2023-02-02

**Authors:** Anush Nayak, S Ve Ramesh, Neetha I R Kuzhuppilly, Vijaya H Pai, Aditya Chaitanya

**Affiliations:** 1 Department of Optometry, Manipal College of Health Professions (MCHP), Manipal Academy of Higher Education (MAHE), Manipal, Udupi (Dist), Karnataka, 576104, India; 2 Department of Allied Health Sciences,Faculty of Life & Allied Health Sciences(FLAHS), Ramaiah University of Applied Sciences, Bangalore, Karnataka, 560054, India; 3 Department of Ophthalmology, Kasturba Medical College, Manipal, Manipal Academy of Higher Education (MAHE), Manipal, Udupi (Dist), Karnataka, 576104, India

**Keywords:** Intraocular pressure, Glaucoma, Goldmann applanation tonometry, iCare HOME

## Abstract

**Introduction:** The purpose of this study was to compare iCare HOME (TA022) with Goldmann applanation tonometer and to evaluate the self-tonometry measurements among the Indian population.

**Methods:** Eligible patients underwent iCare HOME training through guided demonstration (verbal, pictorial, video) and practised self-tonometry measures using iCare HOME. Certification for independent iCare HOME measure was provided if first iCare HOME intraocular pressure (IOP) measurement fell within ± 5 mmHg of Goldmann applanation tonometer (GAT) measurement which was measured by the trained clinician (principal investigator). Certified participants underwent simulated home self-tonometry measurements using iCare HOME, and agreement with GAT IOP measurements was assessed.

**Results:** Seven of 83 participants (8.43%) failed to complete the study due to difficulty in performing the task, leading to non-certification. Patients who could use the iCare HOME had a mean age of 53 ± 15.55years (53% males; 46% females). Only one in 12 subjects did not qualify to use iCare HOME. The overall mean difference between iCare HOME and GAT was 0.83 mmHg (95%, 3.92 and -2.25). At various pressure ranges, 7-16 mmHg, 17-23 mmHg and >23 mmHg, the mean difference between iCare HOME and GAT was 1.22 mmHg (95%, 4.32 and -1.86), 0.77 mmHg (95%,3.69 and -2.19), -0.11 mmHg (95%, 2.52 and -2.74) respectively. The intra-class correlation coefficient of the iCare HOME device was 0.997(95% CI,0.995-0.998).

**Conclusions:** Patients were able to perform self- tonometry using iCare HOME with good reliability and safety. iCare HOME can be used to address the issue of difficulty in acquiring frequent and diurnal IOP measurements by patients doing self-tonometry from home.

## Introduction

Traditionally, intraocular ocular pressure (IOP) management through various medical and surgical procedures is the key factor in controlling the progression of glaucoma. IOP is not constant throughout the day but is subject to change based on the circadian rhythm of the body and other pathological factors. Therefore, managing the IOP of the patient based on a single IOP measurement from one day every four to six months is not effective in controlling the progression of glaucoma. In routine clinical practice, setting a predetermined target for maximum and range of IOP based on diurnal variation test is the key part of management for stabilizing and delaying progression of glaucoma. ^1^^–^^3^ Continuous measurement of IOP using Goldmann applanation tonometry (GAT) is deferred because of factors like invasive procedures, need for inpatient registration, time requirements, lack of comfort, and required specialized skills and manpower. iCare TA01i rebound tonometer (RBT) (iCare Oy, Vanda, Finland), which works based on the impact-induction principle, is simple to use, and doesn’t require anesthetic drops and slit lamp use. ^4^ However, it was developed for a trained professional and not for self-use by patients. The iCare HOME tonometer (TA022; iCare Oy) designed for self-use by patients at home and for regular monitoring of IOP measurements as well as automatic recording of those readings.

Recent guidelines on post-coronavirus disease 2019 (COVID-19) glaucoma care recommend the use of a rebound tonometer as the best IOP measuring device considering patient safety and reduced contamination risks. Since glaucoma patients fall in the age group that is high-risk for COVID-19 infection, reducing their visits to the hospital only for IOP measurements is warranted. iCare HOME has a great potential for in-home self-monitoring of IOP, and demonstrating its usability in the Indian population is important.

This study aimed to investigate the agreement of IOP measurements between self-measurement with an iCare HOME tonometer and Goldmann applanation tonometry, and to assess the precision and feasibility of the iCare HOME tonometer compared to the Goldmann applanation tonometer among the Indian population.

## Methods

The study was performed at the Department of Ophthalmology, Kasturba hospital, Manipal Academy Higher Education, Manipal, from August 2018 to April 2019. All patients underwent routine comprehensive ocular examination including visual field testing and ultrasound pachymetry. Patients with central corneal thickness (CCT) between 450 to 620 μm were included and pachymetry was performed at the end of the study. Palpebral fissure height (PFH) and Hertel exophthalmometry were also measured at the beginning of the study. Patients with near visual acuity lower than 20/200 (binocular), one functional eye and having poor or eccentric fixation were excluded. We also excluded patients with hearing impairment to the extent that the individual cannot hear and converse with others without an assistive aid and/or sign language, as well as patients with inability to demonstrate proficiency with the iCare HOME tonometer after training and failure to complete certification procedures described in the protocol. Patients with any kind of corneal pathologies, disabling arthritis or limited motor coordination limiting self-handling of the iCare HOME tonometer and any history of glaucoma filtering surgery, as well as cataract extraction from subjects’ eyes within the previous two months were excluded. Written informed consent was obtained from all the eligible patients after an explanation of the nature and consequences of this study, and told they could withdraw from the study at any time. Ethical approval was obtained from the Kasturba Hospital Institutional ethics committee (IEC:137/2018). The study was conducted following the Declaration of Helsinki and the ICH-GCP (E6 R1) guidelines. The study was registered in clinical trial registry India (CTRI number CTRI/2018/07/014818).

Enrolled patients underwent baseline slit-lamp examination and corneal staining grading (Oxford) using a fluorescein strip to review the corneal health. This was followed by iCare HOME tonometer training and certification. Patients were given verbal, pictorial and video instructions explaining the use of iCare HOME tonometer then the principal investigators (AN or AC) demonstrated the use of the device for self-tonometry on himself. After the detailed demonstration, patients were asked to perform the self-tonometry using the iCare HOME in the chosen study eye. The eye meeting the eligibility criteria was included in the study. If two eyes were eligible, the eye with high IOP on baseline screening was included. A new disposable probe was used for each patient. During the training session, investigator supervised the patients to provide feedback and required instructions to enhance the performance. The training session was followed by the certification procedure, which involved five self-tonometry measurements by the patients and one GAT measurement by an investigator. As part of the certification process, the investigator silently observed the patients’ performance in handling and positioning of the device without any instructions to ensure the competency of the patient to do self IOP measurements. Later, corneal stain grading was performed and IOP measurement was obtained using fluorescein strip and GAT by an investigator. To get the certification, apart from good handling and positioning, the average of the five self-tonometry measurements obtained by the patient using iCare HOME had to be within 5 mmHg of the measurement using GAT; the range of the five iCare HOME values obtained by the patient also had to be less than or equal to 7 mmHg. After the training and certification procedure, a safety assessment was done by clinical observations of corneal epithelial staining and the patient’s discomfort or any other complications during the procedure was noted to rule out further complications.

Once the patients were certified for the use of iCare HOME, they were given a 20-minute break. Then, patients were instructed to take five self-tonometry measurements using the iCare HOME independently under simulated home-based environment beginning from device setup, loading the probe, and positioning the device without any direct supervision. The number of attempts required to achieve five successful measurements was recorded for each patient in this home-simulated phase. Patients had to decide whether the measurements obtained from the device were correct or not based on the inbuilt device feedback. A maximum of ten attempts was allowed for each patient to get five successful measurements and patients were excluded from the study if they failed to do so. All successful IOP measurements obtained from the device were stored in memory. Unlike other iCare tonometers, iCare HOME does not display the measured IOP, which helps in reducing the bias. Therefore, both patient and investigator knew the patient's measurements at the end of the study once the device was connected to the computer with installed iCare software. After the five successful IOP measurements were obtained from the iCare HOME, each patient underwent GAT apart from corneal stain grading. The calibration of the GAT device was checked every day of the study before the test patients were recruited. Five GAT measurements were obtained from each patient by the investigator and an average of those five measurements was used. Later, corneal thickness was measured for each patient using pachymetry. Both bi-prism used for IOP measurements and pachymetry probe used for corneal thickness measurements were disinfected every time, using alcohol swabs before and after the measurements obtained from the patients to avoid possible infections. At the end of the study, slit lamp examination was repeated and fluorescein grading was done to assess the health of the corneal epithelium. Patients’ complaints of discomfort or any other complications during the procedure were also recorded to assess any possible adverse effect.

The Oxford grading scale was used to grade corneal staining after the certification procedure and at the end of the study. ^5^ Patient discomfort was graded with the help of a visual analogue scale (VAS). ^6^ Patients were asked to mark the VAS scale based on the discomfort. The scoring was measured with the help of a ruler and was converted into a percentage. Discomfort scoring from 1 to 30 % was graded as 1, 31 to 60 % was graded as 2 and a score higher than 61% was graded as 3. By the end of the study, participants were classified into different eye pressure: 7 to 16 mm Hg, 17 to 22 mm Hg and > 23 mm Hg.

### Statistical analysis

Data were recorded in Microsoft Excel 2016 and analyzed using IBM Statistical Package for the Social Sciences (SPSS) software version 20.0. The Shapiro-Wilk test and Kolmogorov-Smirnov test was used to check the normality of the outcome variables. An independent t-test was used to check the significance of age and central corneal thickness; a Chi-square test was used for gender, study eye and handedness to check the significance between participants who were and were not able to perform iCare HOME. A Bland-Altman plot was used to determine the agreement and Spearman’s ρ correlation coefficient was used to calculate the correlation between iCare HOME and GAT. PFH and exophthalmometric values were analyzed using the Mann Whitney U test as the variables were not normally distributed.

## Results

A total of 83 patients agreed to participate and were recruited for the study. Seven of 83 patients (8.43%) failed to complete the study; four of 83 participants (4.82%) were excluded during simulated home measurements as the number of attempts to achieve five successful measurements were more than 10; two of 83 participants (2.41%) were excluded because self-measured IOP did not agree within 5 mmHg of GAT measurements during certification, and one of 83 participants (1.20%) stopped because of difficulty in using the iCare HOME device during the certification process.

A total of 76 patients completed the study and were included in the final data analysis. The mean age of the participants was 53.12 ± 15.55 years, and the percentage of males and females were 53% and 47% respectively. The majority of the study was done in the right eye 70% and 76% of the participants were right-handed ( Table 1).

**Table 1.** Characteristics of the study participants.

Characteristics	Participants who could use iCare HOME (n=76)	Participants who could not use iCare HOME (n=7)	Total Participants (n=83)	P value
Age in years				
Mean (SD)	53.12 (15.55)	51.29 (23.37)	52.96 (16.17)	0.776
Range	21-80	21-78	21-80	
Gender in percentage				
Males	53	57	53	0.574
Females	46	43	47	
Handedness in percentage				
Right	76	43	73	0.055
Left	24	57	27	
Study eye in percentage				
Right	70	14	65	0.003
Left	30	86	35	
Central corneal thickness				
Mean (SD)	515 (32.6)	497 (32.0)	514	0.141
Range	450-613	460-550	450-613	
Number of iCARE attempts (mean±SD)				
Males	4.34 ± 1.3	10.0 ±7.0	4.65 ± 2.18	
Females	4.51 ± 1.4	9.67 ± 4.9	4.86 ± 2.21	
P- value	0.607	0.953	0.663	
Age range ≤ 50	4.72 ± 1.6	13.0	4.97 ± 2.1	
Age range ≥ 51	4.23 ± 1.1	9.0 ± 5.3	4.63 ± 2.2	
P - value	0.137	0.552	0.49	
Time (minutes) taken for practicing iCare HOME (mean±SD)				
Males	9.66 ± 4.1	17.0 ± 12.5	10.24 ± 5.3	
Females	9.56 ± 4.0	19.3 ± 3.0	10.23 ± 4.6	
P- value	0.919	0.770	0.993	
Age range ≤ 50	8.22 ± 3.9	12.5 ± 10.6	8.47 ± 4.3	
Age range ≥ 51	10.6 ± 3.9	21.0 ± 6.6	11.48 ± 5.0	
P - value	0.011	0.278	0.006	

* Independent –t-test was used for Age, central corneal thickness (CCT), Number of iCare attempts and practicing iCare HOME

* Chi-square test was used for gender, handedness and study eye

### Agreement and correlation between ICare HOME and GAT

**Figure 1.**
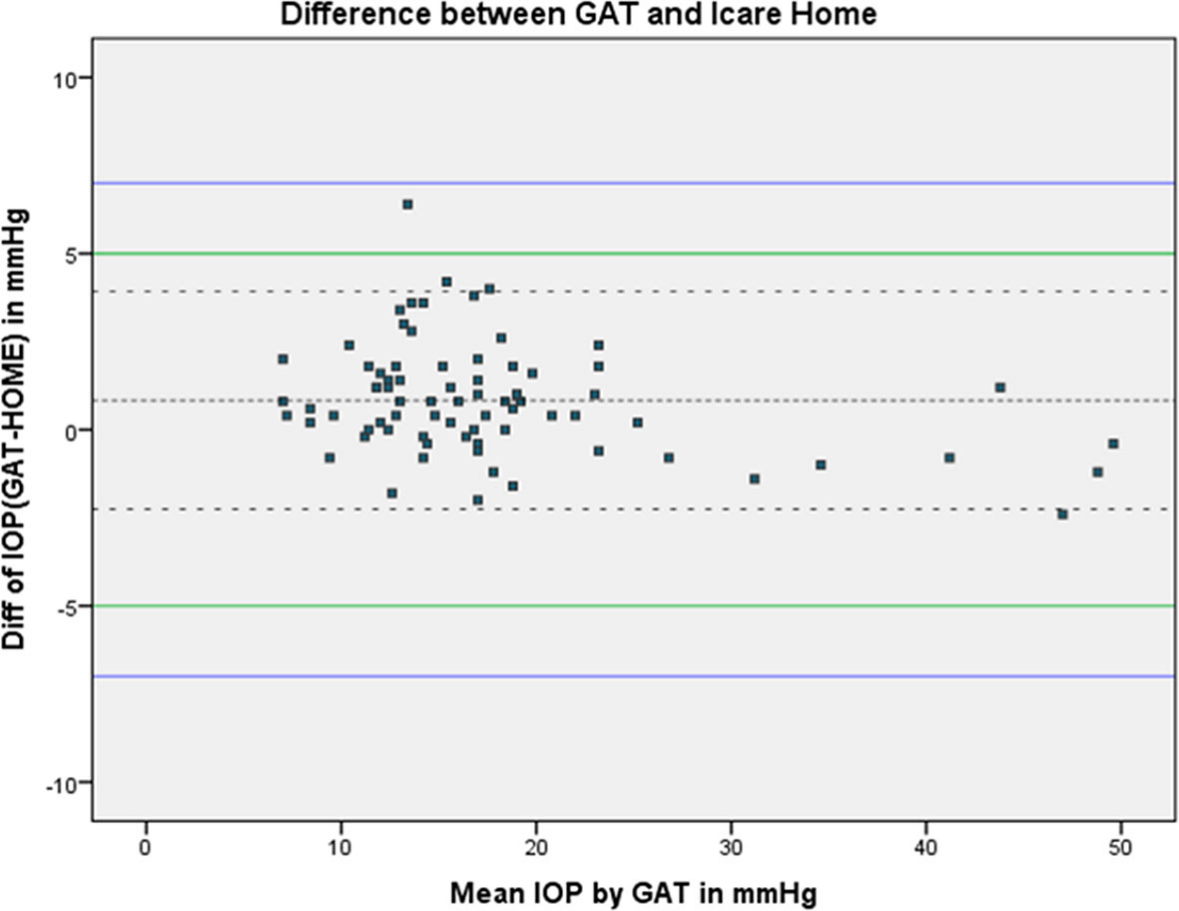
Bland-Altman plot of the agreement between iCare HOME and Goldmann applanation tonometer (GAT).

For 75 of 76 participants (98.6%) iCare HOME measurements agreed within 5 mmHg of GAT. one participant (1.32%) had a difference of more than 5 mmHg of GAT. The overall mean difference between iCare HOME and GAT measurement was 0.83 mmHg with 95% limits of agreement (LOA) between 3.92 and -2.25. Figure 1 shows the Bland-Altman plot of the agreement between iCare HOME and GAT measurements of the patients.

**Figure 2.**
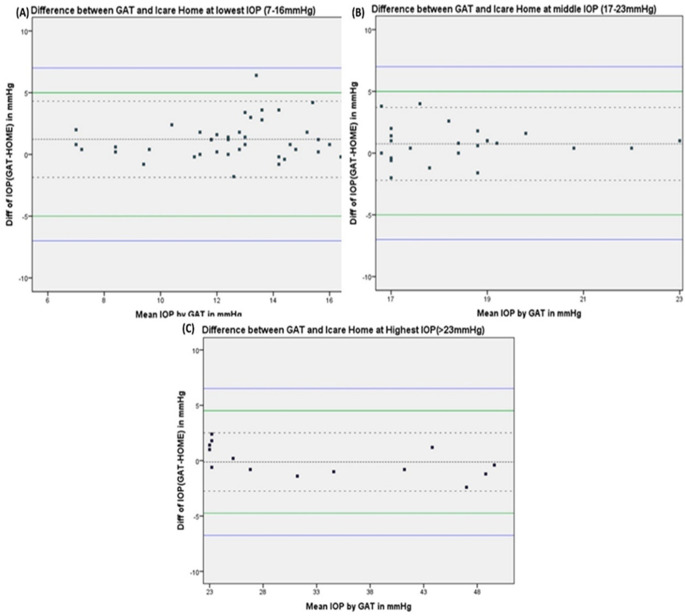
Bland - Altman plot of the agreement between iCare HOME and Goldmann applanation tonometer (GAT) at various ranges of intraocular pressure measurements.

Agreement between iCare HOME and GAT was also assessed at various pressure ranges. At lower IOP (7-16 mmHg) the mean difference between iCare HOME and GAT was 1.22 mmHg with 95% LOA between 4.32 and -1.86. iCare HOME underestimated IOP by more than 5 mmHg in one of 39 participants (2.56%). At the middle IOP range (17-23 mmHg) all the measurements agreed within 5 mmHg with a mean difference of 0.77 mmHg with 95% limits of agreement between 3.69 and -2.19. At a higher IOP range (>23 mmHg) there was a slight overestimation of IOP when compared to GAT measurements with a mean difference of -0.11 mmHg with 95% LOA between 2.52 and -2.74. Figure 2 A-C shows the Bland-Altman plots of agreement between the iCare HOME measurements and GAT measurement over the three different IOP ranges.

**Figure 3.**
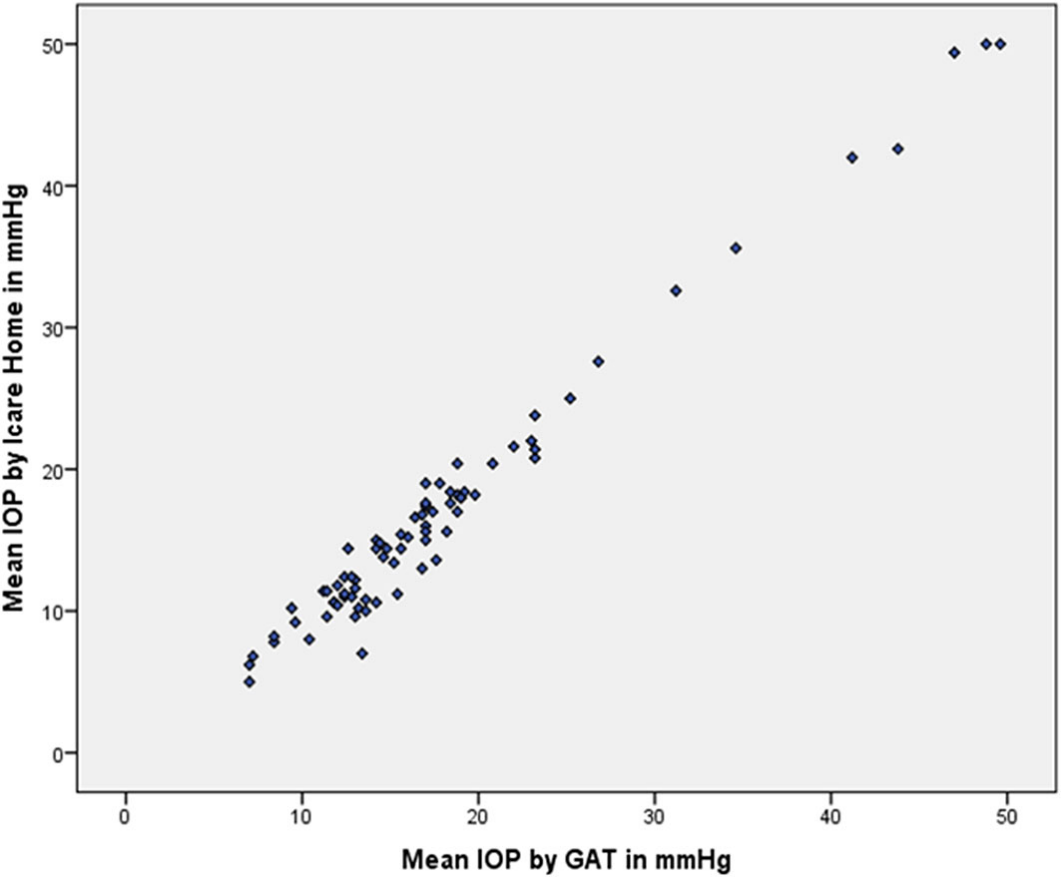
Scatter plot demonstrating the correlation between iCare HOME and Goldmann applanation tonometer (GAT).

Spearman’s ρ correlation coefficient for the association between iCare HOME measurements and GAT measurements was 0.944. The association between the measurements of iCare HOME and the GAT is represented in Figure 3.

### Reliability of iCare HOME measurements

**Figure 4.**
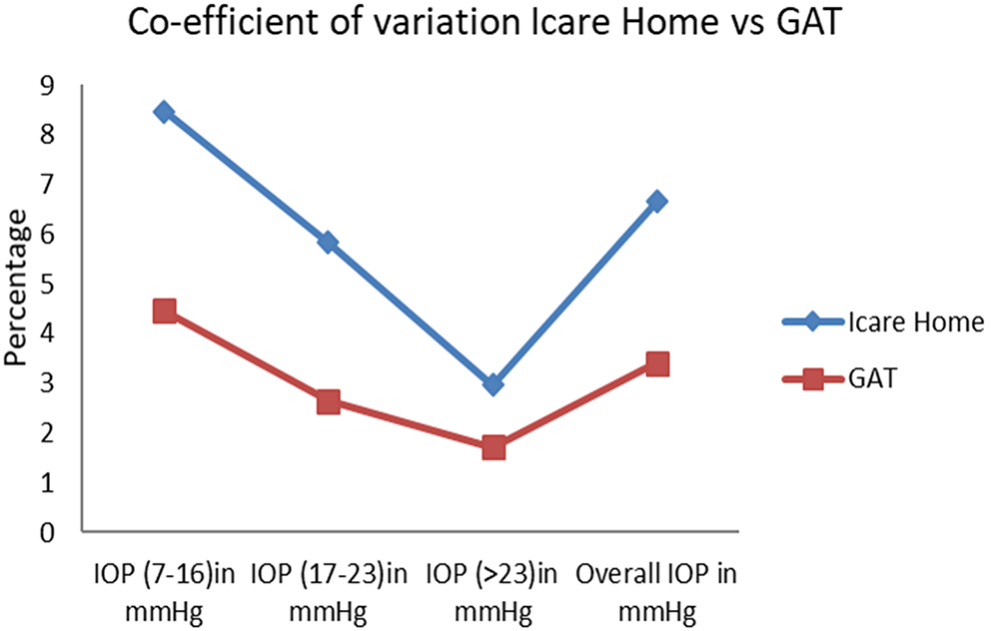
Coefficient of Variation for iCare HOME and Goldmann applanation tonometer (GAT) at various ranges of intraocular pressure measurements.

The reliability of the iCare HOME device was estimated by the co-efficient of variation (COV). Five iCare HOME measurements and five GAT measurements of each participant were analyzed. The COV was assessed separately for each participant for iCare HOME and GAT measurements. The mean COV for each participant was used to find the overall COV for both instruments. The overall COV for iCare HOME was 6.6 and GAT was 3.4. COV for both instruments at various pressure ranges is shown in Figure 4. The precision of iCare HOME was 0.996 (95% confidence interval: 0.994-0.998) calculated by using an intra-class correlation coefficient.

### Comfort and feasibility

**Figure 5.**
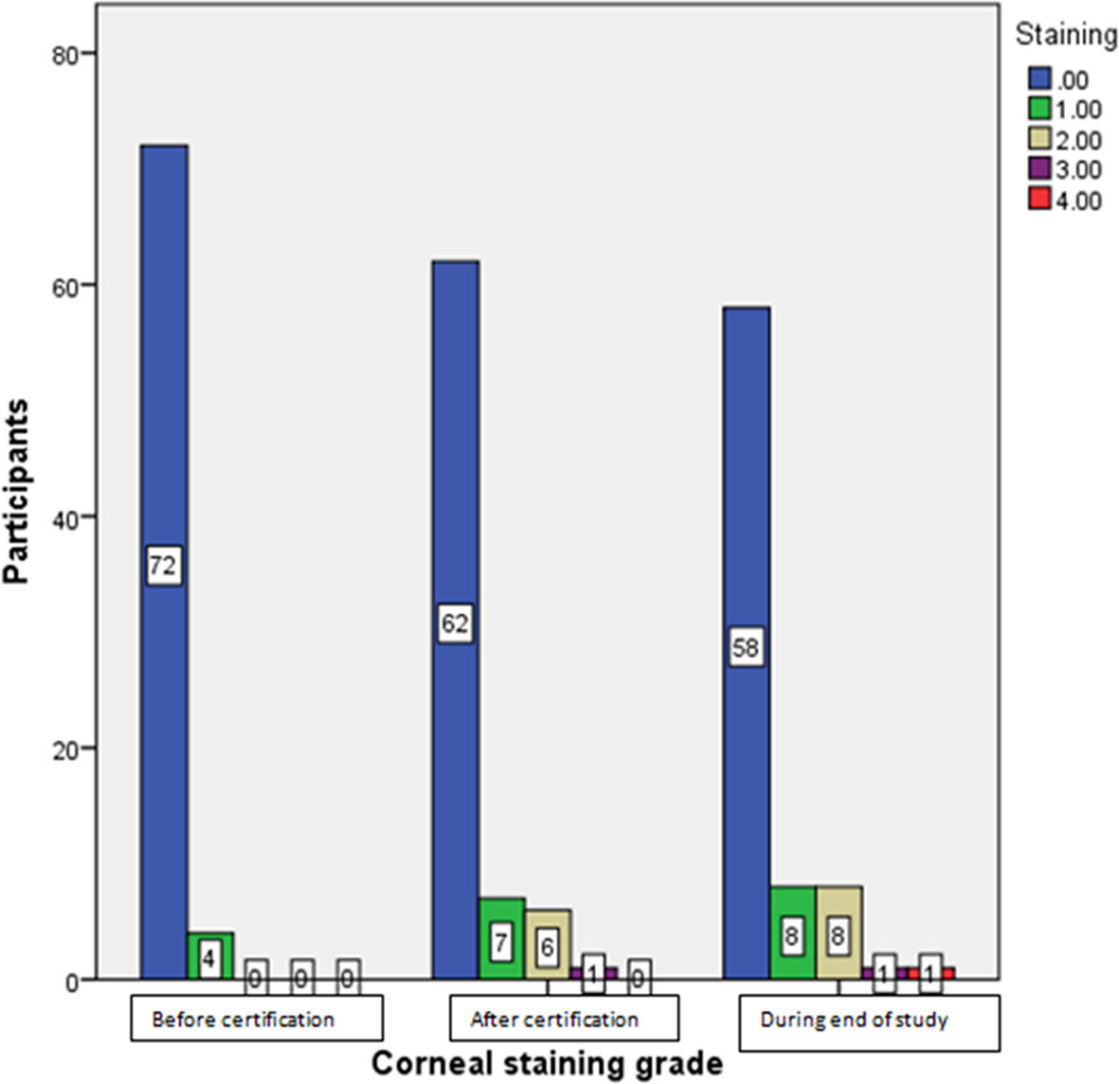
Oxford corneal staining grading after using iCare HOME at different stages of the study.

Comfort scale and corneal staining grade were assessed separately before certification, after certification and at the end of the study. The mean discomfort score before certification was 0.20% which increased significantly to 2.50% during certification. The final score after the simulated home condition was 5.14% with a standard deviation of 12%. None of the patients reported discomfort levels at grade 3 ( Table 2). In 23% of the participants, corneal staining grade became worse, whereas it remained the same in 76% of the participants. Corneal staining grading, grade 1, grade 2, grade 3 and grade 4 were seen in eight, 8,1 and one participants respectively at the end of the study as shown in Figure 5.

**Table 2.** Demonstrating comfort level for iCare HOME at different stages of the study.

Before certification		After certification				During end of the study			
1		VAS scale grade 1		VAS scale grade 2		VAS scale grade 1		VAS scale grade 2	
Participant	%	Participant	%	Participant	%	Participant	%	Participant	%
76	100	74	97.4	2	2.6	73	96.1	3	3.9

### Palpebral fissure height and exophthalmometry

Palpebral fissure height and Hertel exophthalmometer measurement of participants were taken during the baseline examination. The median palpebral fissure height was taken;<10mm was considered a small palpebral fissure and >10mm was considered to be a wide palpebral fissure height. Similarly, the median was taken for Hertel’s exophthalmometry values, Hertel 1 being <17mm and Hertel 2 being >17mm, for analysis. These measurements were compared against the iCare HOME measurements, iCare attempts, time taken for iCare measurement and COV ( Table 3). No significant difference was found between lower and higher PFH and exophthalmometry values.

**Table 3.** iCare HOME findings in real about to with concerning into palpebral fissure height and exophthalmometer.

Parameters	PFH		P value	Exophthalmometry		P value
	PFH 1 (<10mm)	PFH2 (>10mm)		Hertels 1 (<17mm)	Hertels 2 (>17mm)	
iCare Home	14.10mmHg (10.6-18.05)	16.8 mmHg (13.85-19.60)	0.79	14.90 mmHg (11.05-18.4)	15.70 mmHg (10.7-19.8)	0.92
iCare attempts	4 times (3-5)	4.50 times (4-5.75)	0.26	4 times (3-5)	4.5 times (4-5)	0.33
Time taken	10 min (5-12)	12 min (10-13)	0.038	10 min (5-13)	11 min (5.25-14.25)	0.57
COV	0.58 (0.32-0.98)	0.47 (0.29-0.64)	0.12	0.52 (0.30-0.84)	0.57 (0.32-0.93)	0.70

* Results are expressed in Median(Q1-Q3) - Mann-Whitney test

## Discussion

In our study, results showed that iCare HOME self-measuring of IOP can be interchangeably used with GAT. Eligible and certified patients who could perform iCare HOME measurements without supervision, simulating their home environment, showed good agreement (mean difference 0.83 mmHg (95%, 3.92 and -2.25) to the measurements obtained by GAT. This could be used as the best alternative option to conduct a diurnal variation test (DVT) from home in routine clinical practice to monitor glaucoma progression. iCare HOME is also of great benefit to glaucoma care practice in post-COVID care, reducing the number of visits by glaucoma patients to the clinic for IOP examination.

Several studies have reported the accuracy of the iCare rebound tonometer (RBT) in comparison with GAT among different age groups and both in healthy and glaucoma subjects. ^7^^–^^24^ Another iCare self-tonometer, iCare ONE (RT-ONE), also showed good accuracy and agreement with GAT, even in children. ^24^^–^^29^ These studies showed that a higher percentage of patients were able to perform self-tonometry using iCare ONE. ^24^^,^^26^^,^^29^

Similar studies done by Mudie LI *et al*. ^30^ and Dabasia PL *et al*. ^31^ Mudie LI *et al*. ^30^ evaluated the use of iCare HOME in 127 patients with glaucoma or suspected glaucoma. There was a good agreement within 5 mmHg between iCare HOME and GAT in 91.3% of patients with a mean difference of -0.33 ± 3.11 mmHg. Overall, 84% of patients were able to obtain IOP measurements with the iCare HOME within 5 mmHg range of GAT measurements with proper training and certification procedures. The intraclass correlation coefficient for iCare HOME was 0.91 (95% CI, 0.89-0.94). Dabasia *et al*. ^31^ reported an underestimation of IOP by iCare HOME compared to GAT. In this study, 56 (74%) of 76 patients were able to perform self-tonometry with a mean bias of 0.3 mmHg (-4.6 to 5.2) for self-assessment, and 1.2 mmHg (-3.9 to 6.3) for investigator measurement.

Another study by Julia *et al*. ^32^ reported the accuracy of IOP with iCare HOME compared with GAT, and showed a mean IOP difference of -0.8 mmHg (-7.2 to 5.6) between the iCare HOME and GAT. At lower IOP ranges iCare HOME underestimated values, whereas for high IOP it overestimated the measurements compared to GAT. A recent study published by Cvenkel *et al*. ^33^ reported that 88% of patients were able to perform self-tonometry using iCare HOME and 82% of patients fulfilled the requirement for the certification process. The mean (SD) difference in IOP between GAT and iCare HOME was 1.2 (2.4) mmHg (95%, -3.4 to 5.9). This study also reported that 78.5% of patients felt iCare HOME was easy to use and 80.6% of patients expressed their confidence to use the device at the home.

In this study, we validated the iCare HOME among the Indian population. The facial anthropometric differences across ethnic populations and comprehensibility in different populations might influence the reliability of iCare HOME self-tonometry measurements. ^34^ In this study, 91.57% (76) of patients were able to use iCare HOME and fulfilled the certification process. Only 2.41% ( 2) patients were excluded because self-measured IOP did not agree within 5 mmHg of GAT measurements during certification. The certification criteria were adopted as per the best guidelines recommended by the manufacturer. In our study, at a higher IOP range (>23 mmHg) there was a slight overestimation of IOP when compared to GAT measurements with a mean difference of -0.11 mmHg with (95%, 2.52 and -2.74). Mudie *et al*. also reported a slightly higher estimated IOP with iCare HOME for a higher IOP range. Our study also showed good intra-patient reliability of iCare HOME (ICC= 0.996, 95%, 0.994-0.998) similar to reports by Mudie et al. Several previous studies also showed the good reliability of iCare RBT.

In this study, only 3.9% ( 3) patients showed grade 2 level discomfort according to the VAS scale at the end of the study. Also, only two patients showed grade 3 and grade 4 corneal stainings at the end of the study as per the Oxford corneal staining scale. Comparatively, iCare HOME is safer and comfortable to use than GAT. It does not require anesthetic drops and corneal staining to measure IOP as opposed to GAT, making it more comfortable. Demirci *et al*. ^35^ reported that there was no significant difference between GAT and iCare tonometry IOP measurements for all age (7-75) groups; therefore, it is an easy-to-use and reliable alternative to GAT across all age groups. However, iCare HOME cannot be used in a wide range of patients like those with Parkinson’s, arthritis, or dry eyes among others, because of difficulties in handling the tools. In the case of dry eyes, extra precautions may be required as it causes more discomfort. Caregiver can be trained to take IOP measurements for these subsets of patients, however, further research is required to address this issue.

In our study, results showed that 91.57% of the patients were able to perform self-tonometry using iCare HOME with good reliability and safety. There was also a very slight overestimation of IOP with iCare HOME at higher pressure levels. iCare HOME can be used to address the issue of difficulty in acquiring frequent and diurnal IOP measurements by patients doing self-tonometry from home. Therefore, it provides more clinical information to eye care practitioners to monitor and stabilize glaucoma progression.

## Data availability

### Underlying data

Harvard Dataverse: Performance of Home-based self-tonometry (I care HOME (TA022) for measuring intraocular pressure among Normal and Glaucoma patients, https://doi.org/10.7910/DVN/RXRXLS. ^36^

- This project contains the following underlying data: Data entry Master data.xlsx

Data are available under the terms of the Creative Commons Zero “No rights reserved” data waiver (CC0 1.0 Public domain dedication).
