# Supplementary material for: Performance of home-based self-tonometry (iCare HOME (TA022)) for measuring intraocular pressure among healthy and glaucoma patients
Source: F1000Res. 2023 Sep 22;12:128. Originally published 2023 Feb 2. [Version 2] doi: 10.12688/f1000research.123104.2 (PMC11184277; doi:10.12688/f1000research.123104.2)
Supplement: Peer review history

# Reviewer report: Reviewer response for version 2

**DOI:** 10.5256/f1000research.156214.r208647

**Published:** 2024-06-17

**Authors:** Rashima Asokan

**Affiliations:** 1 Elite School of Optometry, Medical Research Foundation chennai, Chennai, India

The authors have incorporated all the suggestions. 

 Can be accepted

Is the work clearly and accurately presented and does it cite the current literature?

Yes

If applicable, is the statistical analysis and its interpretation appropriate?

Partly

Are all the source data underlying the results available to ensure full reproducibility?

Partly

Is the study design appropriate and is the work technically sound?

Yes

Are the conclusions drawn adequately supported by the results?

Yes

Are sufficient details of methods and analysis provided to allow replication by others?

Yes

Reviewer Expertise:

Optometry

I confirm that I have read this submission and believe that I have an appropriate level of expertise to confirm that it is of an acceptable scientific standard.

# Reviewer report: Reviewer response for version 2

**DOI:** 10.5256/f1000research.156214.r263368

**Published:** 2024-04-13

**Authors:** Tiina Pesonen

**Affiliations:** 1 University of Oulu, Oulu, Finland

I want to thank you for the opportunity to read and review this important research article. 

 I have some comment that need to be addressed.

 Introduction

1. There is no description provided regarding the prevalence of glaucoma, its age specificity, or the importance of measuring and receiving treatment. The introduction does not give the reader a complete picture of how many and what kind of people glaucoma usually affects.

Methods

1. You have described what kind of patient you excluded, but I couldn’t find the number of excluded patients. How many patients did you exclude in the first step?
2. Why did you exclude patients with one functional eye, if you only measured eye pressure in one eye?
3. You have excluded patients in many steps during the study. At first step, before certification and after certification. How do you think that affects selection bias?
4. You have examined the agreement of IOP measurements between self-measurement with an iCare HOME tonometer and Goldmann applanation tonometry, but still excluded patients whose results between iCare HOME and Goldmann tonometry differed by of more than 5 mmHg in certification stage. Can you explain how this affects the results.

Results

1. In table 3. Change (N) to N (%)
2. This sentence is unclear to me: “Corneal staining grading, grade 1, grade 2, grade 3 and grade 4 were seen in eight, 8,1 and one participants respectively at the end of the study as shown in “

Discussion

1. The discussion should not be a summary or repetition of the results. Currently there is lot of repetition of results, while it should provide a discussion of the implications for practice. There should also be discussion of selection bias. It seems that patients were able to perform self-tonometry using iCare HOME with good reliability and safety when users are strictly selected. However, glaucoma increases with age. Therefore, most glaucoma patients are older and may also have limitations in using the tonometer independently. Furthermore, older people with physical limitations are also those who may have the greatest challenges in getting to the clinic to measure eye pressure and thus would benefit the most from being able to monitor eye pressure at home. Future studies are needed to find out how reliably iCare HOME tonometry can be used to measure eye pressure in the home environment independently or with the help of a caregiver.

Is the work clearly and accurately presented and does it cite the current literature?

Partly

If applicable, is the statistical analysis and its interpretation appropriate?

Yes

Are all the source data underlying the results available to ensure full reproducibility?

Yes

Is the study design appropriate and is the work technically sound?

Yes

Are the conclusions drawn adequately supported by the results?

Partly

Are sufficient details of methods and analysis provided to allow replication by others?

Partly

Reviewer Expertise:

Optometry, Health Sciences, Vision impairment, especially among older people

I confirm that I have read this submission and believe that I have an appropriate level of expertise to confirm that it is of an acceptable scientific standard, however I have significant reservations, as outlined above.

# Reviewer report: Reviewer response for version 1

**DOI:** 10.5256/f1000research.135174.r162323

**Published:** 2023-05-02

**Authors:** Rashima Asokan

**Affiliations:** 1 Elite School of Optometry, Medical Research Foundation chennai, Chennai, India

The study has an impact in testing the usability of the ICare Home tonometer by subjects with glaucoma. The study has a well designed methodology which was explained in detailed in the manuscript. The authors have taken efforts in minimizing the expected bias in the study. 

 I have only few comments which needs to be addressed by the authors:

 In the methodology classification, IOP was grouped differently than what was shown in the results. Clarify the range appropriately

 Results first section, it is not clear with the total no of participants. If you are explaining about the 7 participants reasons for not included be specific and do not state as out of 83 again and again as it creates confusion for the reader. 

 While describing the participants age in the results section, the mean age of 76 subjects were given whereas in table for 83 participants were given. What is the justification for this?

 In table 1, number of icare attempts, mean and SD was used, it would be better represented as Median and IQR

 Table 1 should also include IOP values for paticipants measured using GAT and Icare.

 Bland altman was represented well in figure 1 and 2. In Figure 2, A B C description should be in detail in the legend

 Scatterplot Figure 3, shows IOP of 50. What were the range of IOP 

 Table 2, representation can be modified. Difficult to process

 Figure 4, COV, Overall IOP to be removed. 

 Table 3 - Legends to be incorporated. 

 Discussion was well elaborated.

Is the work clearly and accurately presented and does it cite the current literature?

Yes

If applicable, is the statistical analysis and its interpretation appropriate?

Partly

Are all the source data underlying the results available to ensure full reproducibility?

Partly

Is the study design appropriate and is the work technically sound?

Yes

Are the conclusions drawn adequately supported by the results?

Yes

Are sufficient details of methods and analysis provided to allow replication by others?

Yes

Reviewer Expertise:

Optometry

I confirm that I have read this submission and believe that I have an appropriate level of expertise to confirm that it is of an acceptable scientific standard, however I have significant reservations, as outlined above.

# Response

**Published:** 2023-09-18

**Authors:** Aditya Chaitanya

**Affiliations:** Manipal acafemy of Higher Education, India

Dear Madam,

 Greetings!

 We thank you for your thoughtful and constructive review of our paper. Your reviews have helped us make important additions and changes to our manuscript (outlined below). We have added these changes in the latest submitted version of article.  Kindly please go through it.

 With kind regards,

 Aditya Chaitanya (on behalf of all authors).

 _____________________________________

 Response to Comments:

 1. In the methodology classification, IOP was grouped differently than what was shown in the results. Clarify the range appropriately

**It is typo error. Group -2 range has been changed from 17-22 mm Hg to 17 -23 mm Hg in methodology.**

 2. Results first section, it is not clear with the total no of participants. If you are explaining about the 7 participants reasons for not included be specific and do not state as out of 83 again and again as it creates confusion for the reader. 

**Modification:**

**“A total of 83 patients agreed to participate and were recruited for the study. Seven of 83 patients (8.43%) failed to complete the study. Among those seven patients, four (4.82%) were excluded during simulated home measurements as the number of attempts to achieve five successful measurements were more than 10, two participants (2.41%) were excluded because self-measured IOP did not agree within 5 mmHg of GAT measurements during certification, and one participant (1.20%) stopped because of difficulty in using the iCare HOME device during the certification process.”  - same mentioned in the results section of the new version.**

 3. While describing the participants age in the results section, the mean age of 76 subjects were given whereas in table for 83 participants were given. What is the justification for this?

**In the table-1 we have given mean age for both 76 subjects and 83 participants**.

 4. In table 1, number of icare attempts, mean and SD was used, it would be better represented as Median and IQR 

**We have provided Median and range for number of iCare attempts in table – 1 as per your suggestion.**

 5.Table 1 should also include IOP values for participants measured using GAT and Icare.

**We have provided IOP values of participants measured using both GAT and iCare Home in newly added table – 2 as per your suggestion.**

 6. Bland altman was represented well in figure 1 and 2. In Figure 2, A B C description should be in detail in the legend

**We have Modified both Figure -1 and 2 as per your suggestion**

 7 **. **Scatterplot Figure 3, shows IOP of 50. What were the range of IOP 

**IOP range in normal participants is from 5 to 20.4 using iCare Home and 7 to19.8 using GAT. In glaucoma patients it ranges from 7 to 50 using iCare Home and 10 to 50 using GAT. IOP range has been added as per your suggestion in Table -2.**

 8. Table 2, representation can be modified. Difficult to process

**We have Modified table to understand easily as per your suggestion.** **This table renamed as Table-3.**

 9. Figure 4, COV, Overall IOP to be removed.

**We have Modified figure-4 as per suggestion.**

 10. ****Table 3 - Legends to be incorporated.

**Modification: "iCare Home findings comparison between two different groups of Palpebral fissure height and Exophthalmometry." This table renamed as Table-4.**

# Reviewer report: Reviewer response for version 1

**DOI:** 10.5256/f1000research.135174.r162324

**Published:** 2023-03-14

**Authors:** Bhaskaran Mani, Sujatha V

**Affiliations:** 1 Glaucoma Services, Sankara Nethralaya, Chennai, Tamil Nadu, India

General

 Authors looked at ICARE Home and suggest the readings can be interchageable with GAT and diurnal tests need to be done using the ICARE Home as self tonometry by patients.

 The Bland Altman test and other results in the study like COV, indicate greater variation and interchangeablility is only moderate. In this context, does the conclusion still hold good?

 Specific

1. Abstract:  Conclusion - "iCare HOME can be used to address the issue of difficulty in acquiring frequent and diurnal IOP measurements by patients doing selftonometry from home". This is not studied, Preferable to say agreement with GAT or not. Then suggest diurnal study comparisons may be warranted,
2. Abstract Results: Please provide Bland Altman 95% agreement limit values instead of mean difference and 95% of mean values, it may be misleading.
3. Introduction - "Recent guidelines on post-coronavirus disease 2019 (COVID-19) glaucoma care recommend the use of a rebound tonometer" - Provide the reference for this.
4. Methods: Inclusion criteria: Mention healthy and glaucoma patients - Define both clearly. Provide the break up of the glaucoma cases as proper diagnosis in results.
5. Methods: Patients were provided video instructions - Please provide a link to the same or mention if it is from the manufacturer.
6. Methods: Mention Icae LINK software or otherwise in full with manufacturer name for the first time.
7. Table 1 - Age group has a large SD, please provide the median and range as well.
8. Figures 1 and 2 - Is the X axis should be Mean of Icare Home and GAT, rather than GAT mean only?
9. Figure 2 - Mention N in each subgroup.
  Table 3: Check title and correct sentence.
10. Discussion: Segregate RBT studies and Icare Home studies. Both are different and serve different purposes.
11. Discussion should address the strengths and limitations of your study, besides the LOA in Bland Altmann need to be discussed in the context of repeatability. CCT influence is not studied.

Is the work clearly and accurately presented and does it cite the current literature?

Partly

If applicable, is the statistical analysis and its interpretation appropriate?

Yes

Are all the source data underlying the results available to ensure full reproducibility?

Yes

Is the study design appropriate and is the work technically sound?

Yes

Are the conclusions drawn adequately supported by the results?

Partly

Are sufficient details of methods and analysis provided to allow replication by others?

Yes

Reviewer Expertise:

Glaucoma, IOP, IMaging, Epidemiology

We confirm that we have read this submission and believe that we have an appropriate level of expertise to confirm that it is of an acceptable scientific standard, however we have significant reservations, as outlined above.

# Response

**Published:** 2023-09-17

**Authors:** Aditya Chaitanya

**Affiliations:** Manipal acafemy of Higher Education, India

Dear Sir,

  We thank you for your thoughtful and constructive review of our paper. Your reviews have helped us make important additions and changes to our manuscript (outlined below). We have added these changes in the latest submitted version of article.  Kindly please go through it.

 With kind regards,

 Aditya Chaitanya (on behalf of all authors).

 _____________________________________

 Response to Comments:

 1. Abstract:  Conclusion - "iCare HOME can be used to address the issue of difficulty in acquiring frequent and diurnal IOP measurements by patients doing self tonometry from home". This is not studied, Preferable to say agreement with GAT or not. Then suggest diurnal study comparisons may be warranted,

**Modification:**  

**"Patients were able to perform self- tonometry using iCare HOME with good reliability and safety. iCare HOME shows good agreement with GAT. Further studies with proper home environment will be recommended to study the diurnal comparisons with GAT."**

 2. Abstract Results: Please provide Bland Altman 95% agreement limit values instead of mean difference and 95% of mean values, it may be misleading.

**We have provided the Bland-Altman 95% agreement limit values in both abstract and results sections in the latest submitted version.**

 3. Introduction - "Recent guidelines on post-coronavirus disease 2019 (COVID-19) glaucoma care recommend the use of a rebound tonometer" - Provide the reference for this.

**Modification:**

  ‘ **'Considering the current post-coronavirus disease 2019 (COVID-19) pandemic rebound tonometer will be the best IOP measuring device for patient safety and less contamination risk".**

 4. Methods: Inclusion criteria: Mention healthy and glaucoma patients - Define both clearly. Provide the break up of the glaucoma cases as proper diagnosis in results.   

**Modification:**

**Glaucoma patients inclusion criteria: "Patients with structural glaucomatous optic disc changes that include RNFL defects, loss of neuro retinal rim, disc haemorrhages, disc asymmetry more than 0.2,   with or without corresponding functional visual field changes were categorised as glaucoma".  - same mentioned in the methods sections.**

 5. Methods: Patients were provided video instructions - Please provide a link to the same or mention if it is from the manufacturer. 

**Modification:**

 " **We have made a detailed video showing usage of iCare Home with proper instructions and easy to understand as per the language preferences. This video was approved by the manufacturer to use in iCare Home related studies."  - same mentioned in the methods sections.**

 6.Methods: Mention Icae LINK software or otherwise in full with manufacturer name for the first time.

**Modification:  "http://www.icarefinland.com/products/icare-link/."  -Mentioned this link in the methodology section.**

 7.Table 1 - Age group has a large SD, please provide the median and range as well.

**We have provided Median and range for age group in table - 1.**

 8.Figures 1 and 2 - Is the X axis should be Mean of Icare Home and GAT, rather than GAT mean only?

**Both Figure-1 and Figure -2  Modified as per your suggestion**

 9. Figure 2 - Mention N in each subgroup.

**Mentioned N in each subgroup in Figure-2.**

 10. Table 3: Check title and correct sentence. 

**Modification: "iCare Home findings comparison between two different groups of Palpebral fissure height and Exophthalmometry." This table renamed as Table-4.**

 11.Discussion: Segregate RBT studies and Icare Home studies. Both are different and serve different purposes.

**Slightly modified as per your suggestion. Second paragraph of the discussion section initially discusses about RBT studies and its reliability. Latter part, discusses about iCare ONE studies. Rest of discussion section particularly discusses about iCare Home studies.**

 12. Discussion should address the strengths and limitations of your study, besides the LOA in Bland Altmann need to be discussed in the context of repeatability. CCT influence is not studied.

**Modification:**

**Limitations: " One of the  limitations of the study is that we have performed this study, i.e. validation of iCare Home, in a simulated home environment at hospital set up. Even though each patient underwent proper training and had a simulated home environment, it may not produce the same results as regular home environment. It also does not address the  learning effect that comes from use over a period of time. So, the future studies should focus on validation of iCare home through a real time home/work place environment. It helps to understand both regular short term and long term diurnal fluctuations of IOP of the patients."  - same mentioned in discussion section .**
